# Improving access to psychosocial interventions for common mental health problems in the United Kingdom: narrative review and development of a conceptual model for complex interventions

**DOI:** 10.1186/1472-6963-12-249

**Published:** 2012-08-13

**Authors:** Linda Gask, Peter Bower, Jonathan Lamb, Heather Burroughs, Carolyn Chew-Graham, Suzanne Edwards, Derek Hibbert, Marija Kovandžić, Karina Lovell, Anne Rogers, Waquas Waheed, Christopher Dowrick, AMP Research Group

**Affiliations:** 1 Manchester Academic Health Science Centre, Health Sciences Research Group, University of Manchester, Manchester, 5th Floor Williamson Building, Oxford Road, M13 9PL, UK; 2 Institute of Psychology, Health and Society, University of Liverpool, Liverpool, UK; 3 Manchester Academic Health Science Centre, School of Nursing, Midwifery and Social Work, University of Manchester, Manchester, 5th Floor Williamson Building, Oxford Road, M13 9PL, UK; 4 Lancashire Care NHS Foundation Trust, Lancashire, UK

## Abstract

**Background:**

In the United Kingdom and worldwide, there is significant policy interest in improving the quality of care for patients with mental health disorders and distress. Improving quality of care means addressing not only the effectiveness of interventions but also the issue of limited access to care. Research to date into improving access to mental health care has not been strongly rooted within a conceptual model, nor has it systematically identified the different elements of the patient journey from identification of illness to receipt of care. This paper set out to review core concepts underlying patient access to mental health care, synthesise these to develop a conceptual model of access, and consider the implications of the model for the development and evaluation of interventions for groups with poor access to mental health care such as older people and ethnic minorities.

**Methods:**

Narrative review of the literature to identify concepts underlying patient access to mental health care, and synthesis into a conceptual model to support the delivery and evaluation of complex interventions to improve access to mental health care.

**Results:**

The narrative review adopted a process model of access to care, incorporating interventions at three levels. The levels comprise (a) community engagement (b) addressing the quality of interactions in primary care and (c) the development and delivery of tailored psychosocial interventions.

**Conclusions:**

The model we propose can form the basis for the development and evaluation of complex interventions in access to mental health care. We highlight the key methodological challenges in evaluating the overall impact of access interventions, and assessing the relative contribution of the different elements of the model.

## Background

The current paper describes the development of a conceptual model of access to care for common mental health problems in the United Kingdom. We outline the importance of access to quality improvement in mental health, and summarise current problems in access. We highlight the importance of conceptual models in the design and evaluation of interventions, and present a synthesis of current models of access of relevance to common mental health problems in the United Kingdom. We apply this synthesis to published interventions to improve access, present a model, and discuss limitations, future development and further evaluation of the model.

### Quality improvement in mental health in the United Kingdom

Health improvement agencies like the National Institute for Health and Clinical Excellence in the United Kingdom recommend interventions for mental health problems such as depression and anxiety because they have demonstrable effectiveness in improving outcomes for individual patients [1]. Even innovative models that attempt to move beyond a focus on the individual patient towards a population perspective still largely seek to achieve their effects through improving outcomes of patients who are already receiving care. For example, the population-based model described by Katon and colleagues focuses on improving quality through enhancing antidepressant adherence among diagnosed patients [2].

### Problems in access to mental health care

Although effectiveness evidence is a critical basis for quality improvement in mental health, many individuals with high levels of mental distress are disadvantaged because of poor *access*, either because care is not available, or because their interaction with care-givers deters or diverts help-seeking into ways that do not meet their mental health needs. Providing comprehensive improvements to quality of care requires addressing both access and effectiveness [3].

**Figure 1:**
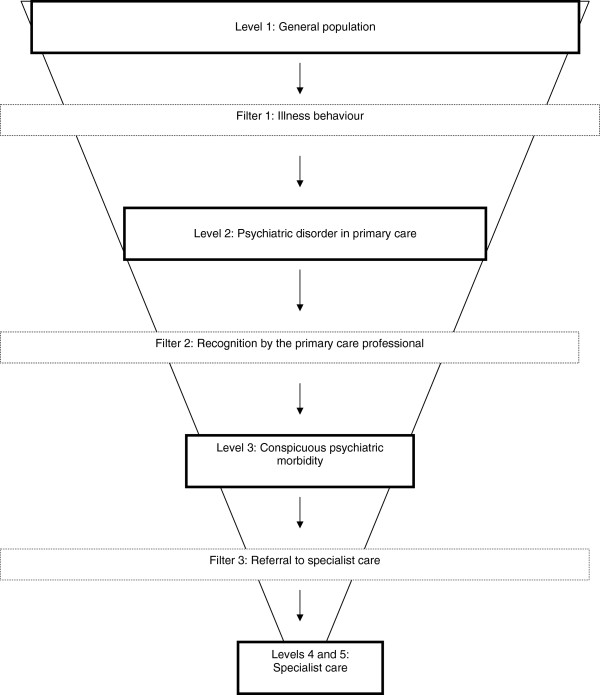
**Pathways to care model **[4-6].

However, in the United Kingdom, policy interest in access to mental health care has largely been restricted to two major issues. Understanding the delivery of mental health care has often been predicated on the influential ‘pathways to care’ model (see Figure 1) [4,5]. This model identifies several ‘filters’ that exist between patients with mental health needs in the community and different ‘levels’ of care, and has highlighted the importance of the recognition of mental health problems by primary care ‘gatekeepers’ [6] and the difficulties of training professionals to improve recognition and referral [7]. The second major policy concern is around the provision of non-pharmacological treatments such as psychological therapy. Although such treatments are generally more acceptable to patients than medication [8], access is far more restricted because of limitations in the number of adequately trained therapists who can deliver these treatments [9].

Developing interventions to improve access to mental health care is a policy priority and has led to two major policy innovations in the United Kingdom. The first has involved payments to general practitioners to use standardised screening instruments to detect cases of depression in populations such as patients with long-term conditions [10], thus increasing the permeability of filter 2 in the ‘pathways to care’ model. The second is the ‘Improving Access to Psychological Therapies’ scheme, which has involved recruitment of psychological therapists [11], the introduction of innovative ‘minimal’ psychological interventions such as computerised treatments [12] and the adoption of the ‘stepped care’ model to maximise patient access to care [13], which has the function of increasing capacity at level 4 and 5 and thus increasing the flow of patients through filter 3.

### Limitations in current understanding of access in mental health

These access innovations are based on assumptions which are not always made explicit. Importantly, there is a focus on *supply-side factors*, such as the availability of treatments and on structural and organisational changes required to reduce or remove the ‘filters’ or other organisational barriers to care. There is generally less explicit consideration of *demand* issues and the factors governing the journey of the patient in need. We argue that such a focus can only partially overcome access issues in the population. Access in mental health is more complex and problematic than in physical health because of issues such as perceived stigma and potential coercion associated with help seeking for mental health problems [14].

Among those involved in intervention development in health services, there is increasing recognition of the need to base the development of interventions and quality improvement activities on conceptual models, such as the Chronic Care Model for quality improvement in the management of long-term conditions [15]. According to current guidance, this involves identifying and developing *theory* and a process of *modelling*. Although the exact nature of these processes has not been clearly defined, the fundamental need is to identify more clearly the rationale underlying the intervention, and the process by which the intervention is expected to lead to relevant outcomes, based on published conceptual and empirical work and new primary studies if required [16].

In this paper we describe a narrative review of current concepts underlying patient access to mental health care, and outline the development of a model of quality improvement in access to care based on these concepts, designed for application in the United Kingdom. We then consider the implications of the model for the development and evaluation of interventions to improve access to mental health care for patients generally, and for poorly served groups such as ethnic minorities and elderly patients. We focus on common mental health problems such as depression and anxiety, on access to care at the interface between the community and primary care, and on access to psychosocial interventions, as these are the areas where access difficulties are most prevalent.

## Methods

Standardised systematic review techniques are effective in synthesising evidence about discrete interventions for use with specific populations but are less applicable when the aim is to synthesise broader concepts. Our review is best conceptualised as the ‘development’ stage in the Medical Research Council framework for complex interventions [16,17]. This stage includes the identification of relevant theories, and the modelling of the key concepts of relevance to an intervention, to allow more effective delivery of the intervention and evaluation of its outcomes. We sought to synthesise published conceptual work on access to mental health care and to use the resulting synthesis to plan the delivery and evaluation of interventions in access to mental health care in selected localities of the United Kingdom.

We undertook an initial scoping search of electronic databases. This included a scoping search of the Cochrane Library using the keyword ‘access’, complemented by a search of MEDLINE, CINAHL, PsycINFO and EMBASE using a search strategy developed in a previous conceptual review [18]. The search terms are shown in Additional file 1. We used our literature searches to identify key ‘models’ of access in the literature. We identified models of relevance to access through our literature search (led by PB, HB and LG) and extracted details from these papers on core concepts. The results of that search were synthesised alongside other sources of evidence, including reviews of the grey literature, dialogues with local stakeholders and secondary analysis of existing datasets [3]. We used facilitated small-group techniques including all authors to develop a preliminary integrated conceptual model of access, drawing on wider work from this programme of research [19-21].

As part of this wider programme of work, we conducted a separate, parallel systematic review to identify interventions to improve access to mental health care in particular exemplar groups, including ethnic minority patients, asylum seekers and refugees, homeless people, adolescents with eating disorders, patients with mild to moderate mental health problems at risk of long-term sickness absence, people with medically unexplained symptoms, patients with depression in advanced cancer, and older patients [3]. To provide a preliminary test of our conceptual model, we explored the relationship between our model and the access interventions identified in our parallel review, to test the utility of our synthesis in both describing existing interventions and providing insights into further developments in this area. Finally, we present an integrated quality improvement model for access to mental health care and explore the challenges in implementing and evaluating such a model.

## Results

### Models of access

Access in a health services context is a complex concept. Proxy measures such as service use or supply of health professionals have been used, but a full consideration of access includes multiple dimensions, such as *adequacy of supply*; *barriers to access*; *effectiveness* (i.e. access to satisfactory health outcomes); and *equity* for different population groups (Table 1) [22].

**Table 1:** **Four core dimensions of access **[22]


Adequacy of Supply	If services are available and supply is adequate, then patients have the opportunity to obtain health care. In this situation a population may 'have access' to health care.
Barriers to Access	‘Gaining access’ may be dependent on barriers, including financial, organisational and social or cultural barriers, as well as adequacy of supply.
Effectiveness	Services must be clinically effective if access to health care is to lead to access to ‘satisfactory health outcomes'.
Equity	Equity may be measured in terms of availability, utilisation or outcomes. Both horizontal (equal treatment for equal groups) and vertical (unequal treatment of unequal groups) equity require consideration.

As noted in the introduction, previous analyses of the management of mental health problems through primary care have focused on the epidemiology of problems in the community and the rates of recognition within primary care, with the assumption that many problems reflect limitations in the skills, knowledge and attitudes of professionals. In response, there has been a focus on supply side issues and structural and organisational change. However, such approaches are less able to account for variations in access to care and the particular problems encountered by groups with poor access such as older patients [23], ethnic minorities [24] and homeless patients [25].

The ambiguity of medical and psychological symptoms and conflicting perceptions of the appropriateness of help-seeking mean that access to care for mental health problems is often characterised by patient uncertainty, which leads to a focus on *illness behaviour (*filter 1 in Figure 1). Illness behaviour has been defined as ‘the varying ways in which individuals respond to bodily indications, how they monitor internal states, define and interpret symptoms, make attributions, take remedial actions and utilise various sources of formal and informal care’ [26].

There are a number of different models of illness behaviour. For example, individualistic psychological models focus on rational cost-benefit calculations, perceptions of barriers and motivation for health care [27], while ‘social barrier’ models highlight health service structure and payment systems and their influence on health care use and decision-making [28]. However, both broadly accept the psychiatric model of mental health and focus on professional sources of care. In contrast, ‘sociological’ [29] and ‘patient-oriented’ [30] models focus on the patient in interaction with professionals and systems, and challenge assumptions about the definition of mental health problems. For example, social network models highlight the influence of those networks as platforms for information and support for decisions whether to access care [29], and also encourage a less static view of access, away from individual and situational ‘determinants’ toward an examination of the *process* of access, over time and in context.

### Process models of access

**Figure 2:**
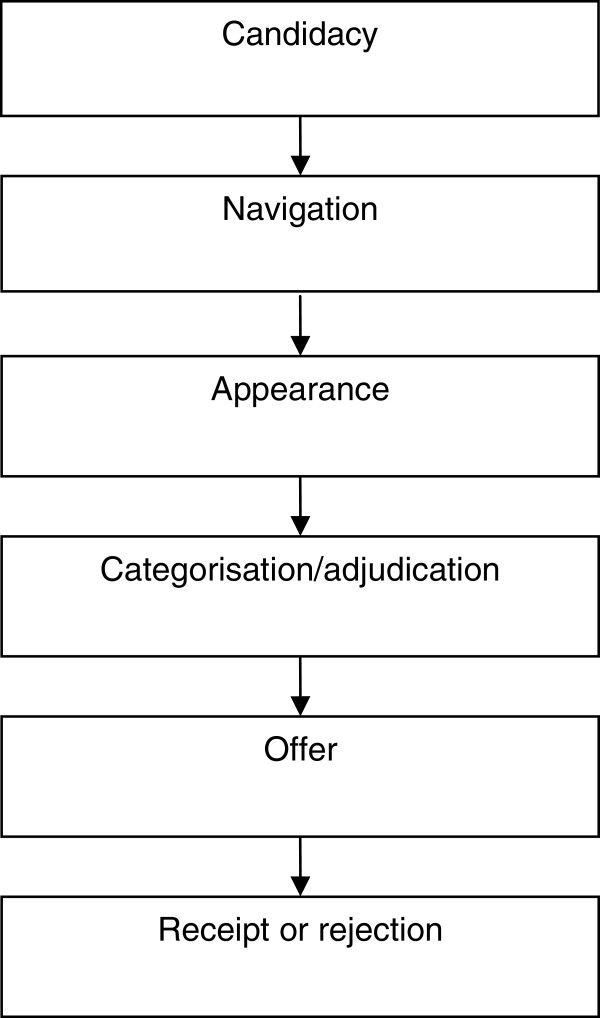
**A process model of access to care (from **[31]**).**

Intervening to improve access requires an understanding of the process by which access is achieved. The ‘filters to care’ model represents one such process model, although it provides limited detail about the processes impacting on movement between levels. Process-orientated models of access identify different stages in access (e.g. system entry, ongoing use) and describe core factors or processes that determine movement into, through and out of the system of care. A recent model based on a critical interpretive synthesis of access by vulnerable groups (but not specific to mental health) describes a process based on five core concepts (Figure 2) [31]. We consider this model in the specific area of access to mental health below.

As noted earlier, help-seeking and access to care for mental health problems is often characterised by uncertainty about the meaning of symptoms and the availability of help. For example, failures to seek treatment are related to a lack of knowledge about common mental health problems and doubts about the effectiveness of treatment [32], neither of which may be influenced by supply-side solutions. The concepts of ‘explanatory models’ [33,34] or ‘illness perceptions’ [35] have been used to capture differences between patient and professional concepts of illness and treatment, and to highlight variation between patient groups in how they understand and respond to illness. The model described in Figure 2 identifies *candidacy* as a core issue, which is defined as ‘how people's eligibility for healthcare is jointly negotiated in interaction between individuals and health services’, and as a ‘dynamic and contingent process, constantly being defined and redefined through interactions between individuals and professionals’. A core concept of relevance to candidacy is *identity*, which refers to an individual’s sense of self, maintained in interaction with others. People are motivated to seek confirmation of their identity in interaction, and there is evidence that negative experiences with health services reflect in part threats to identity [36]. This may involve perceived stereotyping, disempowerment, and feelings that their subjective experience is ignored. The impact of identity may be magnified because of the moral character of help-seeking and access, especially in mental health. Issues of ‘appropriateness’ permeate the access literature, and there is evidence that patients are sensitive to judgments made about their help seeking and perceptions that it may be irrational or wasteful [19,37], which may lead to presentations that are tentative, partial, or delayed, although the evidence is not straightforward [38]. Another strand of the identity discourse deals with *roles*. If illness threatens competence to perform social roles, it may involve a fundamental challenge to self. For example, in women presenting with depression in primary care, perceived failures of competence in primary social roles were interpreted as a sense of duty to seek care [39] but framing help-seeking as a moral action coloured women’s experiences of care, which in turn came to be seen in terms of ‘self-sacrifice’ and the ‘moral dilemma’ of accepting medication. This highlights that access to care provides potential benefits, but is also associated with material and immaterial costs [20]. Understanding how those costs and benefits are perceived and judged is crucial to understanding how candidacy is defined.

Following determination of candidacy, individuals undertake *navigation* to gain a point of entry to health services. This involves a series of psychological and cognitive competencies and resources which include *self efficacy*[40] and *health literacy*[41,42]. These capacities are partly patterned by individual characteristics, but also by the interaction between those individual capacities and the characteristics of the health care system [43]. For example, patients may struggle to make sense of and navigate through services which are labelled, designed and organised around conventional psychiatric and psychological models of mental ill health and which assume concordance between patient and professional concepts [19].

‘Appearances’ can involve a number of different approaches, including appearing before health services through patient-initiated actions, or through *invitations* (where people respond to health services) or *grabs* (where candidacy is not under patient control, such as compulsory hospital admission). Screening for depression during routine medical consultations (as encouraged by current United Kingdom policy) can reflect both ‘invitation’ and ‘grab’ depending on how the approach is perceived by the patient, and there is evidence that both patients and professionals are having to adapt to more proactive forms of ‘appearance’ that these innovations create [10]. Other work has highlighted the role of more diffuse approaches (described as ‘*muddling through’)* in the presence of ambiguous mental health and other symptoms, where the effects of close social networks is emphasised [29].

*Adjudication* refers to professional judgments about the presentation of an individual for intervention or service, influenced by categorisations of patients made by professionals with reference to current services and relationships. Traditionally, this has been dominated by work on recognition and diagnosis and the application of standardised diagnostic systems. There has been controversy about the relevance of conventional psychiatric classification systems in primary care [44-46]. However, decisions about access to services do not simply reflect recognition or non-recognition of symptoms or disagreements about diagnosis - wider considerations are also at play. For example, research has suggested that practitioners’ estimates of the patients’ capacity to benefit from psychological therapy is key [47]. However, little is known about how such judgments are made, and there have been concerns that psychological therapy services have been preferentially delivered to certain populations, threatening equity of access [11], although the relative importance of patient and professional barriers is not known. As psychological therapy services are generally oversubscribed, they may be withheld or rationed in ways which may not be made explicit. Alternatively, inappropriate contact may be negotiated to preserve a relationship with a patient. Although the introduction of systematic approaches to delivery of mental health care such as stepped care and standardised assessment instruments might be expected to reduce variation, evidence suggests that standardised instruments are not yet a key basis for decisions about antidepressant prescribing, and largely seen as secondary to the potential idiosyncrasies of ‘clinical judgment’ [10]. However, there has been relatively little recent research on these complex processes of adjudication.

Adjudication leads to an *offer* (or non-offer) of a health service, which may be accepted or rejected. The current model of improving access in the United Kingdom focuses on increasing patient throughput and the efficiency of treatment delivery, and is based on the assumption that an offer will be sufficient, without necessarily giving enough attention to the likely response. Even innovative sites demonstrating a commitment to improving access to care still show significant levels of failure to engage with available services. For example, of more than 3000 patients in the Doncaster ‘Improving Access to Psychological Therapies’ service who were referred, deemed potentially suitable and completed their involvement with the service, over one quarter attended no sessions. Of these failures to engage, only a third represented mutual agreement between patient and professional. Modern psychological therapy services in the United Kingdom are often of a particular nature (focussed on cognitive-behaviour therapy, delivered via the telephone, and highly structured), and despite the advantages of such an approach, there is developing evidence that significant numbers of patients do not find them in line with their current preferences and needs [48]. Primary care access can be seen as a distinctive field of activity and *habitus*[49], a set of dispositions that generate practices and perceptions of the way in which people encounter it. As primary care presents a set of technologies and relationships, past experience of illness and service contact coalesce with immediate decision-making about use. The concept of *recursivity* captures how the response of the system to patients may reinforce or discourage future health actions [30].

### Potential solutions to problems in access

On the basis of the concepts highlighted in the previous section, we will now move on to potential solutions. We first describe the application of the ideas from the review to published access interventions. We then present a model for quality improvement in access, which draws on the synthesis of reviewed literature and our wider empirical investigations [3]. Finally, we consider the limitations of the model, and consider how its validity and utility can be assessed.

### Interventions to improve access in mental health care

How do published interventions to improve access in underserved groups relate to the model of access described in the previous section? Our systematic search of the literature on access interventions in exemplar groups identified two conventional approaches to improving access:

*Interventions that use existing mental health interventions with underserved populations*. This is in essence the ‘default’ position from effectiveness studies, where models developed for working adult populations (such as ‘collaborative care’) are transposed to groups with difficulties in access such as older people [50] and ethnic minorities [51]. The model discussed so far suggests that this approach will have significant limitations, although there are few empirical demonstrations to allow estimates of the true impact.

*Modification of existing interventions to make them more ‘acceptable’ to poorly served populations* e.g. developing culturally sensitive psychological therapies for low income African-American women [52]. The costs of such modification are expected to yield commensurate benefits, but it is less clear whether those benefits are manifest in increased effectiveness of interventions in those who access care, improved rates of access among those who might not access conventional services, or both.

Genuine access innovations (i.e. those designed to increase the number of people from under-served populations receiving care) are less prevalent although there are individual examples (see Additional file 2). Some of these interventions potentially address a number of the processes outlined in Figure 2. For example, one intervention addressed negotiation of candidacy, navigation and ultimately receipt of care through an intervention in which Community Health Workers used popular education to identify and address health disparities in Latino and African American communities [53]. These workers met regularly with community members to identify health needs and rank intervention priorities.

However, within access innovations in the published literature there was generally insufficient linkage between the content of the intervention and relevant conceptual models, highlighting the importance of development work in the Medical Research Council framework which could provide a relevant ‘line of argument’ about the link between barriers to access and the content of relevant interventions.

### Increasing access to mental health care: key components of an intervention

What are the implications of our review for the development of access interventions in mental health? Like other models of quality improvement such as the Chronic Care Model [15], we propose a complex, multi-level intervention to address:

the world *beyond* primary care, in order to address the processes that occur before service contact (e.g. *candidacy, navigation, appearance*)

the interface with primary care, to address the process by which services and patients agree on appropriate access to care (*categorisation, adjudication and offer)*

the acceptability of interventions available in that setting and the likelihood that they will be attended and used as expected (*receipt)*

**Figure 3:**
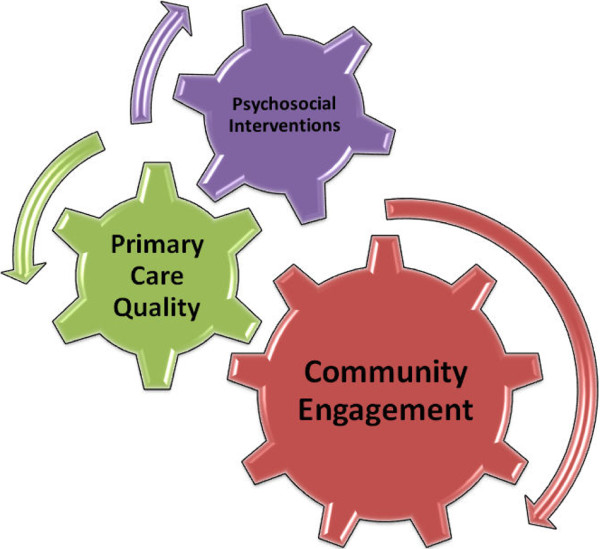
Multifaceted model to improve access to mental health care.

This indicates the need for three major components of a multifaceted model, working synergistically across community and organisational boundaries (see Figure 3).

### Community engagement

Healthcare policy highlights the need for community engagement to ensure sustainable health gains at population level by involving local community stakeholders in improving service delivery. Community engagement or community development has been defined as ‘building active and sustainable communities based on social justice, mutual respect, participation, equality, learning and cooperation. It involves changing power structures to remove the barriers that prevent people from participating in the issues that affect their lives’ [54]. This is done by establishing partnerships with local voluntary organisations, while acknowledging existing stakeholder networks which have developed interventions to address needs. Agencies work with the community providing facilitation, training, support and access to resources and assist them in improving health literacy. In developing sustainable responses, utilizing existing resources and building capacity, this provides continuity beyond the active intervention phase.

The process of community engagement as a component of an intervention to improve access to mental health care should increase patient and public involvement in processes related to mental well-being and empowerment, and potentially improve self efficacy and health literacy [55]. Our earlier description of process models of access highlighted the importance of candidacy. Although there is clearly a role for increasing knowledge and health literacy about interventions for mental health, another role of community engagement would be to help community members judge that the expected benefits of treatment outweigh potential material and immaterial costs of accessing services [20], and thus potentially enhancing judgments of their ‘candidacy’ for intervention. This may extend beyond issues of knowledge and health literacy to more complex processes around perceptions of identity and roles, where far less is known about possible interventions. As noted earlier, judgments of candidacy are also dynamic and contingent, and the relative contribution of variables such as knowledge, identity and roles is also likely to reflect the particular context in which the population and intervention is situated.

The most efficient and effective way of increasing access is likely to be through working with agencies already established within the community who will already be providing some input to the target groups, and work with them in considering what are the particular areas of met and unmet need that might be addressed by a new intervention. Knowledge exchange among stakeholders should enhance health care providers’ awareness of existing community resources, enable understanding of timely, effective, appropriate, respectful and acceptable treatment, and facilitate the other two streams of the model in making such services available. However, there is also a need to recognise that there will be people that these agencies are less successful in serving. There is a risk of offending the personnel of agencies that have considerable experience and local credibility. However, these agencies may not be working successfully together, or with other sectors such as general practice or local mental health services. There is a need to give healthcare providers information about the community in forms which they can readily assimilate into their day-to-day practice. Personal presentation by people with whom the target community identifies is a potent element in fostering and maintaining engagement. This leads onto the second level of the model – addressing the quality of primary care.

### Addressing quality of primary care

To improve patient experience at the interface with services, and to ensure that the processes of *categorisation, adjudication* and *offer* are managed appropriately, health care teams need to increase competence in responding to *appearances,* taking account of local context [19]. Training content needs to be negotiated with practice staff to meet their identified needs, with focus on populations and groups they have particular concerns about. The intervention also needs to include local user views about issues such as the importance of continuity and proximity of care, problems with mental health terminology, and structural and organisational barriers to access, including the built environment and reception by front-line staff. This demonstrates the link with the community engagement level, and highlights the important role of services in exacerbating existing difficulties in mental health literacy.

Notwithstanding innovations in the delivery of mental health care, a fundamental requirement for patients is to feel they have been ‘listened to’ [19,56]. This requires engagement with patient explanatory models [33], and addressing issues of *cultural competence,* through clinicians taking account of patient values, beliefs and practices (which may or may not reflect the patient’s membership of a particular group). Cultural competence can be seen as a specific form of patient-centredness, where the clinician ‘tries to enter the patient's world, to see the illness through the patient's eyes’ [57]. Cultural competence requires identification of ‘what is at stake in local worlds’ in relation to the everyday lives of patients [58]. Implementing cultural competence has been hampered by lack of conceptual clarity, a failure to distinguish between organisational and individual competence, lack of awareness of the influence of professional values on response to suffering, and a tendency to associate culture solely with race and ethnicity. Clinicians need to develop a ‘shared narrative’ with patients: perceptions of appropriate behaviour in responding to illness can be embedded in such narratives, the underlying values of which are situated in wider contexts. Professionals should understand the patient’s ‘helper model’ i.e. the patient’s conceptualisation of the role of the professional (and others) in management [59].

Interventions for cultural competence include recruiting staff who reflect population diversity; interpretation and language services; staff cultural awareness training; language appropriate healthcare materials; and culturally specific healthcare settings. However evidence for the efficacy of these interventions is limited. A review found good evidence that cultural competence training could impact on professional knowledge, attitudes and skills, some evidence of impact on satisfaction, but sparse evidence of impact on adherence or patient outcomes [60].

The preceding analysis is based on an assumption that the processes of categorisation, adjudication and offer are relatively well understood and amenable to change through educational interventions. These assumptions are open to challenge. As noted earlier, relatively little is known about how professionals categorise patients in terms of their mental health needs and adjudicate access to mental health care, and even if these processes are well understood, evidence that education leads to changes in practitioner behaviour and patient outcomes in primary care mental health is not consistent [7,61]. Although a range of quality improvement activities in primary care are likely to be critical, there is an argument that certain populations may be better served by *removal* of primary care as gatekeeper for access, precisely because the process of categorisation, adjudication and offer are idiosyncratic and not easily amenable to change. Evidence from the ‘Improving Access to Psychological Therapies’ sites in the United Kingdom suggests that self referral may have functioned as a factor improving access among patients from ethnic minorities [11]. In this case, a combination of community engagement and provision of tailored psychosocial interventions may have functioned to overcome barriers raised by the quality of primary care, at least for some populations.

### Providing tailored psychosocial interventions

There is evidence for the effectiveness of tailored psychosocial interventions for some under-served groups, particularly older people [62] and ethnic minorities [3]. Interventions should probably be tailored to meet the personal and communal needs of those who may benefit from them, although there may be a trade-off between tailoring and cost-effectiveness [63]. Some tailoring may be limited in scope – for example, modifications of CBT interventions to increase their acceptability while remaining broadly in line with conventional delivery [64]. A recent trial of depression treatment in South Asian women utilised a more innovative social intervention designed to maximise acceptability [24], but there is a tension between modifications to enhance access and ensuring that core, evidence-based mechanisms of action are not diluted or lost. Tailoring should be based on an understanding of the psychosocial factors that permeate participants’ lives, and influence their response to an intervention. At a basic level, the use of the term ‘mental health’ is unacceptable to many groups: other terms (such as ‘*wellbeing’*) [65] could increase engagement. Group and face to face sessions may be of variable acceptability between groups depending on helper models and perceived identity threats associated with public discussions about mental health care. However, our earlier discussions of *candidacy* and related issues of identity and roles highlight the complexity of the processes that might underlie judgments of the acceptability of treatments and the likely response to *offers*. The developing methods of qualitative synthesis may be useful in exploring these issues and providing further insights to allow effective tailoring. Issues of tailoring highlight debates about the role of preferences and choice in access to mental health treatments [66], the tension between providing standardised treatments favoured by the clinical guideline development process, and the role of health services in meeting the personal preferences of patients. There is also the complex question of whether providing interventions in line with preferences is a major determinant of outcome [67].

Attention needs to be paid to interfaces, enhancing linkage with community engagement, and encouraging involvement of local communities and primary care providers in the design of psychosocial interventions. Engagement and recruitment may be enhanced by case managers, actively linking service users into the intervention and with other key providers from primary care according to the principles of collaborative care [51].

## Discussion

### Summary

This paper presents a conceptual model based on a process model of access to care and incorporating interventions at three levels. The levels comprise (a) community engagement (b) addressing the quality of interactions in primary care and (c) the development and delivery of tailored psychosocial interventions.

### Limitations of the review

The narrative review was designed to support the delivery and evaluation of complex interventions in access to mental health care in the United Kingdom. We applied systematic search methods as far as possible, but it is difficult to provide a clear and transparent description of the synthesis to allow replication, and standardised quality assessment of papers included in the review is problematic. The model has been designed to have maximum relevance in the United Kingdom context, although many of the core processes are likely to generalise more widely. However, financial barriers to access (other than those governing overall level of service provision) are notably absent, whereas they may be critical in other health care contexts such as the United States. However, there may be contextual issues within the United Kingdom which will have implications for the operation of the model, such as variation in current quality of care in different locations, or the impact of existing variation in community attitudes to mental health.

We restricted the testing of our model in terms of its ability to describe existing access interventions. Further testing of the utility and validity of our model are discussed below.

### Implications of the model

Most research into improving access in mental health care has lacked a clear conceptual model, and failed to address systematically the different elements of the patient journey from decisions about candidacy to receipt of service. There is a danger that the impact of current interventions and policy developments in access to care will be limited. We propose that our synthesis of existing work provides a useful framework for the development of integrated approaches to access interventions in mental health.

Of course, the challenge for those who propose complex, multi-level models is to develop ways of evaluating them, capturing both process and outcome and enabling the interactions between levels to be assessed.

As noted previously, the development of the model has been undertaken from a health services research perspective which seeks to place the development of interventions on a sounder conceptual basis [16], with a view to eventual experimental or quasi- experimental evaluation. In this context, maximum impacts would be expected when an intervention encompasses all three levels of the model, and when interventions at each level are functioning in concert. However, given the likely increase in costs associated with such multi-faceted interventions, their relative importance is a crucial research question. For example, can tailored interventions and improved quality of delivery in primary care improve population health, or does community engagement function as a necessary ‘effect multiplier’ of the other aspects of the model? Are certain populations better served by focussing on community engagement and the development of tailored interventions and the *replacement* of primary care as a key part of the referral process? The same questions are faced by other quality improvement interventions such as the Chronic Care Model, where the relative importance of different facets of the model in determining outcomes is only now beginning to be assessed [68].

**Figure 4:**
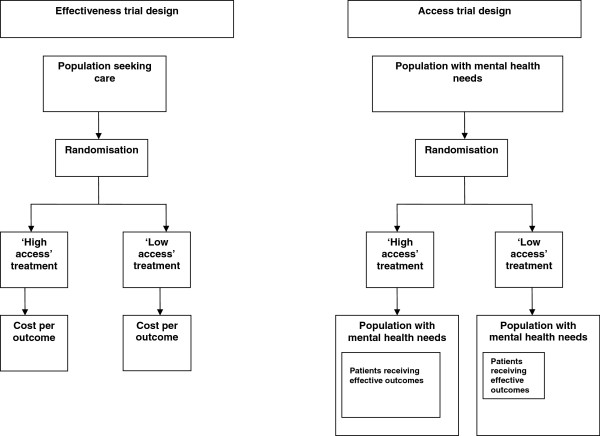
Access trial designs.

One of the major problems is that there are few studies of the impact on innovations on access *per se*, as the evaluation is complex. Access effects are not readily amenable to conventional trial designs (Figure 4), because those designs generally take a population of patients accessing care and randomise them between different services, assessing average effectiveness and cost-effectiveness in those who have already agreed to receipt of services. Assessments of ‘minimal interventions’ (such as self-help and computerised self-help treatments) and suchlike which demonstrate that less resource-intensive services can achieve equivalent clinical outcomes [69]*imply* potential benefits in access, because more effective treatments can be provided from equivalent resource. However, the potential to achieve greater population access to care is not formally tested, and the cost per outcome benefits found in the population of patients accessing care may not scale up to those in the wider population. A rigorous assessment of the effect of an access intervention would involve assessment of mental health care in a population of patients before and after the introduction of an access intervention, compared to a population who did not receive that intervention. Outcome assessment would involve calculation of both the absolute numbers from a target population who successfully negotiate the stages of the model in Figure 1 to receive appropriate high quality care, the distribution of care according to need, and the effectiveness of that care. Although there are examples in the literature that approach this design [7,70,71] and designs which might be able to accommodate it [72] we did not locate any studies that provided a comprehensive assessment of access, equity and effectiveness. It is likely that exploration of these issues will require mixed methods research, combined with enhanced use of routinely collected data from public health and provider sources, and creative use of comparators for intervention sites [73,74]. Such an approach should enable an adequate assessment of whether this comprehensive approach to service re-design improves access to primary care mental health.

Of course, experimental and quasi-experimental evaluation have important limits, in terms of practicality, ethical limits, and the ability to isolate single causal mechanisms in the context of ‘open’ systems [75]. Other criteria for the utility of the model relate to what has been described as *authenticity*[76]. This may involve assessment of the ability of the model to help stakeholders to make sense of access issues in new and useful ways. Additionally, it may involve what has been described as *catalytic authenticity*, which is defined as the ‘extent to which action is stimulated and facilitated by the evaluation process’ [76]. The current model is part of a large research programme that seeks to both understand access problems and generate specific solutions for use in a local contexts [3]. We intend to use extensive work with study participants and local stakeholders in services and communities to understand whether the model has functioned in such a way to help them overcome the problems they have identified and to generate new and useful solutions.

## Conclusions

In this paper, we describe a conceptual model that may be of utility in the development and evaluation of complex interventions to improve access to mental health care. We have described the key methodological challenges in evaluating the overall impact of access interventions, and suggest a critical next step is to explore the relative contribution of the different elements of the model and the ways in which they interact in practice.

## Competing interests

The authors declare that they have no competing interests

## Authors’ contributions

Literature searching was conducted by PB, HB and LG. All authors contributed to the development of the paper, read and approved the final manuscript.

## Pre-publication history

The pre-publication history for this paper can be accessed here:

http://www.biomedcentral.com/1472-6963/12/249/prepub

## Supplementary Material

Additional file 1 Search strategy (November 2007, Medline, CINAHL, Psycinfo, EMBASE). Click here for file

Additional file 2 Access innovations in the literature. Click here for file
