# Supplementary material for: Improving access to psychosocial interventions for common mental health problems in the United Kingdom: narrative review and development of a conceptual model for complex interventions
Source: BMC Health Serv Res. 2012 Aug 13;12:249. doi: 10.1186/1472-6963-12-249 (PMC3515797; doi:10.1186/1472-6963-12-249)
Supplement: Additional file 1 — Search strategy (November 2007, Medline, CINAHL, Psycinfo, EMBASE). [file 1472-6963-12-249-S1.doc]

**Additional file 1 Search strategy (November 2007, Medline, CINAHL, Psycinfo, EMBASE)**

| Models, theoretical/ |
| --- |
| what is.m_titl. |
| the meaning of.m_titl. |
| taxonomy.tw. |
| typology.tw. |
| conceptuali?ation.tw. |
| conceptually.tw. |
| (theor$ adj1 map$).tw. |
| (theor$ adj1 model$).tw. |
| (theor$ adj1 framework$).tw. |
| (theor$ adj1 meaning$).tw. |
| (theor$ adj1 approach$).tw. |
| (theor$ adj1 overview$).tw. |
| (theor$ adj1 feature$).tw. |
| (theor$ adj1 (mode or modes)).tw. |
| (theor$ adj1 characteristic$).tw. |
| (theor$ adj1 form?).tw. |
| (conceptual$ adj1 map$).tw. |
| (conceptual$ adj1 model$).tw. |
| (conceptual$ adj1 framework$).tw. |
| (conceptual$ adj1 meaning$).tw. |
| (conceptual$ adj1 approach$).tw. |
| (conceptual$ adj1 overview$).tw. |
| (conceptual$ adj1 feature$).tw. |
| (conceptual$ adj1 (mode or modes)).tw. |
| (conceptual$ adj1 characteristic$).tw. |
| (conceptual$ adj1 form?).tw. |
| (defin$ adj1 map$).tw. |
| (defin$ adj1 model$).tw. |
| (defin$ adj1 framework$).tw. |
| (defin$ adj1 meaning$).tw. |
| (defin$ adj1 approach$).tw. |
| (defin$ adj1 overview$).tw. |
| (defin$ adj1 feature$).tw. |
| (defin$ adj1 (mode or modes)).tw. |
| (defin$ adj1 characteristic$).tw. |
| (defin$ adj1 form?).tw. |
| ((theor$ or conceptual$ or defin$) adj1 (scheme or schema)).tw. |
| (literature adj1 analys?s).tw. |
| Combined with: |
| (access adj5 care).mp. |
