# Supplementary material for: Improving access to psychosocial interventions for common mental health problems in the United Kingdom: narrative review and development of a conceptual model for complex interventions
Source: BMC Health Serv Res. 2012 Aug 13;12:249. doi: 10.1186/1472-6963-12-249 (PMC3515797; doi:10.1186/1472-6963-12-249)
Supplement: Additional file 2 — Access innovations in the literature. [file 1472-6963-12-249-S2.doc]

**Additional file 2**

Access innovations in the literature

| 1. Interventions to affect decisions about *candidacy* and help-seeking e.g. educating South Asian women about depression and its treatment [1]  2. Interventions designed to increase the likelihood of *appearance* for treatment e.g. a session of motivation enhancement therapy to address readiness to change for people with eating disorders [2]  3. Interventions designed to increase the likelihood of *invitations* and *adjudication* by health care professionals e.g. emergency room screening and structured assessment of older people at risk of functional decline [3]   1. Technological and delivery design interventions to increase access such as treatment delivery via telephone or internet [4,5] or through community groups [6] |
| --- |

Reference List

1. Jacob K, Bhugra D, Mann A: **A randomised controlled trial of an educational intervention for depression among Asian women in primary care in the United Kingdom.** *Int J Soc Psychiatry* 2002, **48:**139-148.

2. Feld R, Woodside D, Kaplan A, Olmsted M, Carter J: **Pretreatment motivational enhancement therapy for eating disorders: a pilot study.** *Int J Eat Disord* 2001, **29:**393-400.

3. McCusker J, Verdon J, Tousignant P, de Courval L, Dendukuri N, Belzile E: **Rapid emergency department intervention for older people reduces risk of functional decline: results of a multicenter randomized trial.** *J Am Geriatr Soc* 2001, **49:**1272-1281.

4. Kaltenthaler E, Shackley P, Stevens P, Beverley C, Parry G, Chilcott J: **A systematic review and economic evaluation of computerised cognitive behaviour therapy for depression and anxiety.** *Health Technol Assess* 2002, **6 (22)**.

5. White H, McConnell E, Clipp E, Branch L, Sloane R, Pieper C, Box T: **A randomized controlled trial of the psychosocial impact of providing internet training and access to older adults.** *Aging and Mental Health* 2002, **6:**213-221.

6. Brown J, Cochrane R, Hancox T: **Large scale health promotion stress workshops for the general public: a controlled evaluation.** *Behavioural and Cognitive Psychotherapy* 2000, **28:**139-151.
